# HLA-A Alleles and the Risk of Cervical Squamous Cell Carcinoma in Japanese Women

**DOI:** 10.2188/jea.JE20090155

**Published:** 2010-07-05

**Authors:** Satoyo Hosono, Takakazu Kawase, Keitaro Matsuo, Miki Watanabe, Hiroaki Kajiyama, Kaoru Hirose, Takeshi Suzuki, Kumiko Kidokoro, Hidemi Ito, Toru Nakanishi, Yasushi Yatabe, Nobuyuki Hamajima, Fumitaka Kikkawa, Kazuo Tajima, Hideo Tanaka

**Affiliations:** 1 Deparment of Gynecology and Obstetrics, Nagoya University Graduate School of Medicine, Nagoya, Japan; 2 Division of Epidemiology and Prevention, Aichi Cancer Center Research Institute, Nagoya, Japan; 3 Department of Epidemiology, Nagoya University Graduate School of Medicine, Nagoya, Japan; 4 Department of Planning and Information, Aichi Prefectural Institute of Public Health, Nagoya, Japan; 5 Department of Medical Oncology and Immunology, Nagoya City University Graduate School of Medical Science, Nagoya, Japan; 6 Department of Gynecologic Oncology, Aichi Cancer Center Hospital, Nagoya, Japan; 7 Department of Molecular Diagnosis, Aichi Cancer Center Hospital, Nagoya, Japan; 8 Department of Preventive Medicine/Biostatistics and Medical Decision Making, Nagoya University Graduate School of Medicine, Nagoya, Japan; 9 Director, Aichi Cancer Center Research Institute, Nagoya, Japan

**Keywords:** cervical cancer, human leukocyte antigen, case-control study

## Abstract

**Background:**

We conducted a case-control study to examine the relationship between human leukocyte antigen-A (HLA-A) allele polymorphism and the pathogenesis of cervical neoplasia among Japanese women.

**Methods:**

A total of 119 patients with invasive cervical squamous cell carcinoma were compared to 119 age- and menopausal status-matched non-cancer controls. Blood samples were taken from all cases and controls and lifestyle information was collected by means of a self-administered questionnaire. The estimated impact of HLA-A alleles on cervical cancer risk was evaluated by unconditional logistic regression models.

**Results:**

The frequency of HLA-A*0206 among cases was significantly lower than among controls (*P* = 0.006). There was an inverse association between A*0206 and cervical cancer risk (odds ratio [OR] = 0.31, 95% confidence interval [95% CI] = 0.15 to 0.65, *P* = 0.002), and a positive association for HLA-A*2402 (OR = 1.76, 95% CI = 1.00 to 3.09, *P* = 0.048). After correction for multiple comparisons, A*0206 was significantly associated with reduced cervical cancer risk (corrected *P* = 0.036). Furthermore, the inverse association between A*0206 and cervical cancer risk was independent of smoking status (never smoker: OR = 0.37, 95% CI = 0.15 to 0.90; ever smoker: OR = 0.23, 95% CI = 0.06 to 0.89).

**Conclusions:**

There was an inverse association between HLA-A*0206 and cervical cancer risk among Japanese women, which suggests that HLA-A polymorphism influences cervical cancer risk. Further investigation in other populations is thus warranted.

## INTRODUCTION

Cervical cancer is the second most common cancer in women worldwide.^1^ Although many epidemiological and laboratory studies have shown that oncogenic human papillomavirus (HPV) is the primary causative agent,^1^^,^^2^ most women with HPV infection do not develop cervical cancer. Other factors, such as environmental co-factors and individual immune response to infection, are therefore presumed necessary to promote persistent HPV infection and the progression of the increasingly severe cervical intraepithelial neoplasia that precedes invasive cancer.^3^^–^^6^ Indeed, several studies have shown a high rate of persistent HPV infection and HPV-associated malignancy in immunocompromised patients.^7^^–^^9^

Recently, much attention has been focused on the biological properties of human leukocyte antigen (HLA) in the clearance of HPV infection and tumor progression in cervical carcinogenesis.^1^^,^^10^ HLA class I molecules, which are expressed in most somatic nucleated cells, are largely responsible for the presentation of pathogen-derived peptides from the cytosol to CD8-positive cytotoxic T-lymphocytes (CTLs), and are also target ligands for killer cell immunoglobulin-like receptors (KIRs). HLA class II molecules, however, are found on antigen-presenting cells and present peptides degraded in intracellular vesicles to CD4-positive helper T cells.^11^ An effective immune response may therefore require optimal peptide presentation by both class I and II molecules to activate effector- and helper-T cell responses. While several epidemiological studies have revealed clinically relevant associations between HLA class I polymorphisms and some viral infections, particularly human immunodeficiency virus infection and the development of AIDS,^12^ the evidence for an association of HLA class I polymorphisms with HPV infection and cervical cancer is insufficient.

The results of studies investigating major histocompatibility complex (MHC) polymorphisms and cervical cancer susceptibility in different populations have been inconsistent,^13^^–^^27^ and most studies of HLA and cervical cancer have focused on HLA class II alleles.^18^^–^^22^ Although the importance of CTL responses to viral infection and tumor regression are well recognized, few studies have investigated HLA class I alleles.^24^^–^^27^

We conducted a case-control study to examine the association between HLA-A alleles and invasive cervical cancer risk in Japanese women. In addition, we evaluated the effect of smoking—an established modifiable risk factor for cervical cancer^3^^,^^28^—on HLA-A alleles and cervical cancer risk.

## METHODS

### Subjects

The cases were 119 patients who had received a histological diagnosis of invasive cervical squamous cell carcinoma and first visited the Aichi Cancer Center Hospital (ACCH) in Japan between January 2001 and November 2005. The controls (*n* = 119) were randomly selected from 11 814 women who were diagnosed as cancer-free and matched with a 1:1 case-control ratio to cases by age (±3 years), referral pattern, and year of first visit to our hospital. All subjects were recruited within the framework of the Hospital-based Epidemiologic Research Program at Aichi Cancer Center (HERPACC), as described elsewhere.^29^^,^^30^ In brief, information on lifestyle factors was collected using a self-administered questionnaire from all first-visit outpatients at Aichi Cancer Center Hospital aged 20 to 79 who were enrolled in HERPACC between January 2001 and November 2005. Patients were also asked about their lifestyle when healthy, or before their current symptoms developed, and a trained interviewer checked all the questionnaires. The questionnaire was completed by 96.7% of eligible subjects and 52.1% provided blood samples for HERPACC. Our previous study showed that the lifestyle patterns of first-visit outpatients accorded with those in a randomly selected sample of the general population of Nagoya City.^31^ The data were loaded into the HERPACC database and routinely linked with the hospital-based cancer registry system to update the data on cancer incidence. All participants gave written informed consent and the Institutional Ethics Committee at Aichi Cancer Center approved the study. All subjects in this study completed the questionnaires and provided blood samples.

### HLA typing

Blood samples were taken from all cases and controls to examine HLA genotyping. HLA genotypes of the HLA-A allele in all cases and controls were identified by the Luminex microbead method (Luminex 100 System; Luminex, Austin, TX) according to the nomenclature of The HLA Dictionary 2004^32^ at the NPO HLA laboratory (Kyoto, Japan). The Luminex method has been proven to type all HLA alleles with a frequency greater than 0.1% in the Japanese population. The Japanese Society for Histocompatibility and Immunogenetics annually assesses the quality of HLA genotyping in the NPO HLA laboratory by conducting re-genotyping using 60 randomly selected samples; they confirmed complete concordance of the results of genotyping. These HLA-typing results were included in the statistical analysis.

### Assessment of lifestyle factors

Smoking status, drinking status, and history of oral contraceptive use were classified into binary categories (never or ever), while gravidity was classified into 3 categories (0, 1–2, ≥3).

### Statistical analysis

To assess the associations between each HLA-A allele and the risk of cervical cancer, odd ratios (ORs) with 95% confidence intervals (CIs) were estimated using unconditional logistic models adjusted for potential confounders. Potential confounders considered in the multivariate analyses were age, smoking status (never or ever), drinking status (never or ever), menopausal status (premenopausal or postmenopausal), gravidity (0, 1–2, ≥3), and history of oral contraceptive use (never or ever). Missing values for any covariate were treated as a dummy variable. Differences between the cases and controls in categorized demographic variables were tested by the chi-square test, and age and gravidity in cases and controls were further compared by the Mann-Whitney test. Stratification analysis was used to estimate risk for subgroups by smoking status (never or ever). A *P* value less than 0.05 was considered statistically significant. A corrected *P* value was obtained by multiplying the *P* value by the number of alleles tested for each locus (Bonferroni correction),^33^ and a corrected *P* value less than 0.05 was considered statistically significant. All analyses were conducted using STATA version 10 (Stata Corp., College Station, TX.).

## RESULTS

The baseline characteristics of the 119 cervical cancer patients and 119 controls are summarized in Table 1. Age, drinking status, menopausal status, gravidity, and history of oral contraceptive use did not differ between the 2 groups. However, a history of smoking was significantly more common in cases (*P* = 0.009).

**Table 1.** Characteristics of subjects

Characteristic	Cases		Controls		*P* value
	(*n* = 119)	(%)	(*n* = 119)	(%)	
Age [median (min-max)], yrs	42 (25–74)		43 (26–73)		0.975
<30 (%)	6	(5.0)	6	(5.0)	0.998
30–39 (%)	46	(38.7)	44	(37.0)	
40–49 (%)	26	(21.9)	28	(23.5)	
50–59 (%)	23	(19.3)	23	(19.3)	
≥60 (%)	18	(15.1)	18	(15.1)	
Smoking status					
Never (%)	69	(58.0)	88	(74.0)	0.009
Ever (%)	50	(42.0)	31	(26.0)	
Alcohol status					
Never (%)	68	(57.1)	61	(51.3)	0.362
Ever (%)	51	(42.9)	58	(48.7)	
Menopausal status					
Premenopausal (%)	35	(29.4)	37	(31.1)	0.844
Postmenopausal (%)	82	(68.9)	82	(68.9)	
Unknown (%)	2	(1.7)	0	(0)	
Gravidity [median (min-max)]	2 (0–10)		2 (0–6)		0.511
0 (%)	20	(16.8)	26	(21.9)	0.622
1–2 (%)	52	(43.7)	48	(40.3)	
≥3 (%)	46	(38.7)	45	(37.8)	
Unknown (%)	1	(0.8)	0	(0)	
Oral contraceptive use					
Never (%)	107	(89.9)	110	(92.4)	0.781
Ever (%)	10	(8.4)	9	(7.6)	
Unknown (%)	2	(1.7)	0	(0)	

The frequencies of 18 distinct HLA-A alleles that were detected in cases and controls are summarized in Table 2. The frequency of A*0206 antigen was significantly lower in cases than in controls (12.6% vs 26.9%, respectively; *P* = 0.006), and while the frequency of A*2402 was higher in cases, the difference was not statistically significant (64.7% vs 54.6%; *P* = 0.113). Table 3 shows the associations between the 18 HLA-A alleles identified in the subjects and cervical cancer risk. The A*0206 antigen showed a significant inverse association (OR = 0.31; 95% CI = 0.15 to 0.65; *P* = 0.002), whereas A*2402 showed a positive association (OR = 1.76; 95% CI = 1.00 to 3.09; *P* = 0.048). After Bonferroni correction, however, the only significant association was between A*0206 and reduced cervical cancer risk (corrected *P* = 0.036).

**Table 2.** Frequencies of HLA-A alleles among cases and controls

HLA-A	Cases		Controls		*P* value^a^

	(*n* = 119)	(% cases)	(*n* = 119)	(% controls)	
A*0101	2	1.7	4	3.4	0.408
A*0201^b^	24	20.2	25	21.0	0.873
A*0206^c^	15	12.6	32	26.9	0.006
A*0207	11	9.2	8	6.7	0.473
A*0218	0	0	1	0.8	0.316
A*0301	1	0.8	0	0	0.316
A*1101^d^	21	17.6	16	13.4	0.371
A*1102	1	0.8	0	0	0.316
A*2402^e^	77	64.7	65	54.6	0.113
A*2407	1	0.8	0	0	0.316
A*2420	2	1.7	3	2.5	0.651
A*2601	12	10.1	15	12.6	0.540
A*2602	4	3.4	8	6.7	0.236
A*2603	3	2.5	4	3.4	0.701
A*2606	0	0	1	0.8	0.316
A*3101^f^	26	21.8	18	15.1	0.182
A*3303^g^	17	14.3	23	19.3	0.298
A*7401	1	0.8	0	0	0.316

^a^chi-square test.

^b^1 homozygote in cases and 1 homozygote in controls.

^c^1 homozygote in controls.

^d^1 homozygote in cases and 1 homozygote in controls.

^e^15 homozygotes in cases and 12 homozygotes in controls.

^f^2 homozygotes in cases.

^g^1 homozygote in cases.

**Table 3.** Associations between individual HLA-A alleles and risk of cervical cancer

HLA-A	Cases (*n* = 119)	Controls (*n* = 119)	Age-adjusted odds ratios (95% CI)	*P* value	Multivariate odds ratios (95% CI)^a^	*P* value
A*0101	2	4	0.49 (0.09–2.74)	0.417	0.50 (0.08–2.96)	0.441
A*0201	24	25	0.95 (0.51–1.78)	0.873	1.05 (0.54–2.05)	0.893
A*0206	15	32	0.39 (0.20–0.77)	0.007	0.31 (0.15–0.65)	0.002
A*0207	11	8	1.42 (0.55–3.67)	0.473	1.38 (0.52–3.71)	0.519
A*0218	0	1	—	—	—	—
A*0301	1	0	—	—	—	—
A*1101	21	16	1.38 (0.68–2.81)	0.370	1.65 (0.77–3.51)	0.195
A*1102	1	0	—	—	—	—
A*2402	77	65	1.53 (0.91–2.59)	0.111	1.76 (1.00–3.09)	0.048
A*2407	1	0	—	—	—	—
A*2420	2	3	0.66 (0.11–4.03)	0.653	0.66 (0.11–4.14)	0.660
A*2601	12	15	0.78 (0.35–1.74)	0.539	0.74 (0.32–1.74)	0.490
A*2602	4	8	0.48 (0.14–1.65)	0.244	0.37 (0.10–1.32)	0.123
A*2603	3	4	0.74 (0.16–3.40)	0.702	0.69 (0.13–3.52)	0.654
A*2606	0	1	—	—	—	—
A*3101	26	18	1.57 (0.81–3.05)	0.183	1.41 (0.70–2.82)	0.334
A*3303	17	23	0.70 (0.35–1.38)	0.300	0.73 (0.36–1.49)	0.389
A*7401	1	0	—	—	—	—

^a^Multivariate models adjusted for age, smoking, drinking, gravidity, oral contraceptive use, and menopausal status.

Table 4 shows the analysis stratified by smoking status. The OR of A*0206 antigen was 0.37 (95% CI = 0.15 to 0.90; *P* = 0.028) among never smokers and 0.23 (95% CI = 0.06 to 0.89; *P* = 0.033) among ever smokers, while a positive association was only observed between A*2402 antigen and ever smokers (OR = 3.43; 95% CI = 1.26 to 9.36; *P* = 0.016).

**Table 4.** Associations between individual HLA-A alleles and risk of cervical cancer, by smoking status

**Never smoker**						
HLA-A	Cases (*n* = 88)	Controls (*n* = 69)	Age-adjusted odds ratios (95% CI)	*P* value	Multivariate Odds ratios (95% CI)^a^	*P* value
A*0101	3	1	0.42 (0.04–4.10)	0.453	0.49 (0.05–4.94)	0.542
A*0201	20	16	1.06 (0.50–2.25)	0.885	1.31 (0.58–2.94)	0.511
A*0206	23	9	0.43 (0.18–1.00)	0.049	0.37 (0.15–0.90)	0.028
A*0207	6	5	1.02 (0.30–3.53)	0.969	1.24 (0.34–4.46)	0.745
A*0218	1	0	—	—	—	—
A*0301	0	1	—	—	—	—
A*1101	14	18	2.01 (0.90–4.47)	0.086	1.95 (0.85–4.48)	0.116
A*1102	0	0	—	—	—	—
A*2402	53	46	1.28 (0.66–2.49)	0.461	1.19 (0.59–2.39)	0.631
A*2407	0	0	—	—	—	—
A*2420	3	1	0.42 (0.04–4.12)	0.453	0.37 (0.04–3.76)	0.399
A*2601	10	6	0.77 (0.26–2.25)	0.633	0.62 (0.19–2.00)	0.424
A*2602	5	2	0.46 (0.08–2.45)	0.360	0.43 (0.07–2.46)	0.343
A*2603	1	3	4.48 (0.45–44.90)	0.202	4.41 (0.41–48.03)	0.223
A*2606	0	0	—	—	—	—
A*3101	11	13	1.64 (0.68–3.95)	0.267	1.65 (0.68–4.04)	0.270
A*3303	16	9	0.65 (0.26–1.58)	0.340	0.65 (0.26–1.62)	0.357
A*7401	0	0	—	—	—	—

**Ever smoker**						
HLA-A	Cases (*n* = 31)	Controls (*n* = 50)	Age-adjusted odds ratios (95% CI)	*P* value	Multivariate odds ratios (95% CI)^a^	*P* value

A*0101	1	1	0.60 (0.04–10.02)	0.724	0.32 (0.01–7.46)	0.480
A*0201	5	8	1.02 (0.30–3.47)	0.981	0.77 (0.20–2.93)	0.673
A*0206	9	6	0.33 (0.11–1.06)	0.063	0.23 (0.06–0.89)	0.033
A*0207	2	6	2.10 (0.39–11.38)	0.388	2.66 (0.44–16.25)	0.289
A*0218	0	0	—	—	—	—
A*0301	0	0	—	—	—	—
A*1101	2	3	0.89 (0.14–5.71)	0.900	0.78 (0.11–5.50)	0.801
A*1102	0	1	—	—	—	—
A*2402	12	31	2.63 (1.04–6.63)	0.041	3.43 (1.26–9.36)	0.016
A*2407	0	1	—	—	—	—
A*2420	0	1	—	—	—	—
A*2601	5	6	0.70 (0.19–2.52)	0.580	0.86 (0.23–3.32)	0.832
A*2602	3	2	0.39 (0.06–2.48)	0.318	0.22 (0.03–1.72)	0.232
A*2603	3	0	—	—	—	—
A*2606	1	0	—	—	—	—
A*3101	7	13	1.20 (0.42–3.45)	0.730	1.04 (0.34–3.21)	0.946
A*3303	7	8	0.62 (0.20–1.96)	0.417	0.75 (0.22–2.61)	0.652
A*7401	0	1	—	—	—	—

^a^Multivariate models adjusted for age, drinking, gravidity, oral contraceptive use, and menopausal status.

## DISCUSSION

In this study, we found an inverse association between A*0206 antigen and the risk of invasive cervical cancer among Japanese women. This inverse association remained after stratification by smoking status. To our knowledge, this is the first study to investigate the association between the risk of invasive cervical cancer and HLA-A alleles in Japanese women.

Our results suggest that HLA-A polymorphism might be a host genetic factor that affects cervical cancer risk by immunological mechanisms. HLA class I molecules present foreign antigenic peptides to CTLs and play a crucial role in the host response to infectious diseases and tumor suppression. HLA class I genes are also important in innate immune response, as HLA class I molecules are ligands for the KIR on natural killer cells, which include both activating and inhibitory receptors, and the interaction between KIR and MHC class I molecules influences resistance to many human diseases.^34^ In addition, loss of HLA surface expression has been observed in various solid tumors and tumor cell lines.^35^ Koopman et al showed that 50% of multiple HLA allele alterations are caused by loss of heterozygosity in the HLA region, which is frequently detected in cervical cancer.^36^^,^^37^ Such alterations in class I antigen expression may enable HPV-infected cancer cells to escape immune system detection, which may in turn be an indicator of the effectiveness of the HLA immune system in tumor surveillance. Given the putative association between various HLA polymorphisms and the risk of cervical neoplasia,^13^^,^^14^^,^^16^^,^^19^^,^^21^^,^^22^ as well as the established biological association between HLA class I molecules and cervical cancer,^37^^–^^40^ the relative lack of epidemiological research on the importance of each HLA class I allele is surprising.^13^^,^^24^^,^^25^^,^^27^ In particular, the results of the few extant epidemiological studies on HLA-A alleles and cervical neoplasia have been inconsistent and controversial, as compared with those investigating other HLA class I alleles.^13^^,^^25^^,^^27^

After adjustment for multiple comparisons we found an inverse association between A*0206 and the risk of squamous cell cervical cancer in Japanese women. The data on A*0206 are strikingly limited. This may be due to the markedly low frequency of this allele worldwide; however, the frequency is higher among Asians and Native Americans than among whites.^41^ The distribution of HLA alleles is known to correlate with ethnicity, and ethnicity is a determinant of disease risk in many populations. Although Wang et al showed a null association between A*0206 and cervical neoplasia among women in Costa Rica (OR = 1.12, 95% CI = 0.36 to 3.43),^27^ Chan et al found an inverse association in Hong Kong Chinese women (OR = 0.08, 95% CI = 0.01 to 0.32).^25^ The latter finding is compatible with our results, and, taken together, these results suggest an association between HLA-A polymorphism and the risk of cervical neoplasia. Although the function of each HLA allele is not fully understood, our findings add new evidence that A*0206 has a role in the pathogenesis of cervical neoplasia.

The inverse association between A*0206 and cervical cancer risk was independent of smoking status, as shown in Table 4. Smoking is an established HPV cofactor in the development of preinvasive and invasive cervical neoplasms.^3^^,^^28^ Although the molecular mechanism by which smoking influences cervical cancer is unclear, there are several plausible hypotheses.^42^^,^^43^ Recently, Nadais et al showed that smoking reduced the number of intraepithelial Langerhans cells in normal uterine cervix next to cervical intraepithelial neoplasia grade 3.^44^ Wiley et al revealed that current smokers were twice as likely as nonsmokers to test positive for HPV16 serum antibody.^45^ These findings suggest the potential for interaction between smoking and the immune system. In contrast, our findings indicate that smoking and the HLA immune system may act on cervical carcinogenesis independently via different pathways. This finding requires further investigation.

Several potential limitations of our study warrant consideration. First, we had no information on HPV status. HPV is a transient infection in most women, a fact that hampers accurate evaluation when examination is only conducted at enrollment. Moreover, an association between sexual behavior and HLA expression is implausible. To mitigate this lack of information on HPV infection, our analysis adjusted for gravidity, smoking, and oral contraceptive use. Second, we were not able to evaluate the linkage with other HLA loci, such as HLA-B, C, DRB1, DQB1, and DPB1. Major HLA haplotypes among the Japanese population, however, do not include HLA-A*0206,^46^ and the distance from the HLA-A locus to other major loci is far, as compared with that between HLA-B and HLA-C or between HLA-DRB1 and HLA-DQB1.^11^ Although the influence of other loci on the effects of HLA-A*0206 is thus likely to be small, we cannot deny the possibility that another critical gene or genes linked with the HLA region might in fact be the causative agent. Regardless, further investigation, such as haplotype analysis or fine mapping of HLA regions using tag SNPs, is needed. Third, as this was a hospital-based case-control study, the comparability of cases and controls depended on whether the cases and controls arose from the same source population. In the ACCH, it is assumed that individuals who have not received a diagnosis of cancer during a particular period of time will visit the ACCH when they do develop a malignancy. We therefore assume that our controls are appropriate for the drawing of causal inferences. Finally, our study had a modest sample size, and replication of our results in populations of differing ethnicity is required.

In conclusion, this case-control study indicates that HLA-A*0206 decreases the risk of invasive squamous cervical cancer among Japanese women, and that this protective effect is present regardless of smoking status. Further investigation of these findings is warranted, preferably with adequate consideration of HPV status, in analyses involving combinations with other alleles.
